# Sociodemographic and delivery risk factors for developing postpartum depression in a sample of 3233 mothers from the Czech ELSPAC study

**DOI:** 10.1186/s12888-017-1261-y

**Published:** 2017-03-21

**Authors:** Adam Fiala, Jan Švancara, Jana Klánová, Tomáš Kašpárek

**Affiliations:** 1 Department of Psychiatry, Masaryk University, Brno, Czech Republic; 2 Research Centre for Toxic Compounds in the Environment (RECETOX), Faculty of Science, Masaryk University, Kamenice 5, 625 00 Brno, Czech Republic; 3 Institute of Biostatistics and Analyses, Faculty of Medicine and Faculty of Science, Masaryk University, Brno, Czech Republic

**Keywords:** Postpartum depression, PPD, Risk factors, ELSPAC, EPDS, Postpartum blues, Mood disorders

## Abstract

**Background:**

In the postpartum period, certain groups of women are at a higher risk for developing depressive episodes. Several studies have described risk factors for developing postpartum depression (PPD). However, these studies have used limited numbers of participants, and therefore the estimated prevalence of PPD varies greatly.

**Methods:**

The objective of this study is to identify the main risk factors for developing PPD by using data collected via the Czech version of the European Longitudinal Study of Pregnancy and Childhood (ELSPAC). This database provides a representative sample (*n* = 7589) observed prospectively and a large amount of data on depressive symptoms and on biological, socioeconomic, and environmental factors.

The Edinburgh Postnatal Depression Scale (EPDS) was used to screen for incidence of PPD. The affective pathology was examined at three time points: before delivery, 6 weeks after delivery, and 6 months after delivery.

**Results:**

The prevalence of depressive symptoms before delivery was 12.8%, 6 weeks after delivery 11.8%, and 6 months after delivery 10.1%. The prevalence rates are based on women who completed questionnaires at all three time-points (*N* = 3233).

At all three time points, the main risk factors for developing PPD identified as significant by both univariate and multivariate analysis were personal history of depressive episodes and mothers experiencing psychosocial stressors. Other risk factors occurring in both types of analysis were: family history of depression from expectant mother’s paternal side (prenatal), mothers living without partners (6 weeks postpartum) and feelings of unhappiness about being pregnant (6 months postpartum). Several protective factors were also observed: male child gender (prenatal), primiparous mothers (6 months postpartum), and secondary education (prenatal, only by multivariate analysis).

Significant risk factors found solely by univariate analysis were family history of depression in both parents of the expectant mother (prenatal and 6 weeks postpartum), family history of depression from subject’s maternal side (6 months postpartum), unintentional pregnancy (prenatal and 6 weeks postpartum), feelings of unhappiness about being pregnant (prenatal and 6 weeks postpartum), primary education (prenatal and 6 weeks postpartum), mothers who opted not to breastfeed (6 months postpartum) and mothers living without partners (prenatal and 6 months postpartum). Family savings were identified as protective factor (prenatal and 6 months postpartum).

**Conclusions:**

We identified significant predictors of PPD. These predictors can be easily detected in clinical practice, and systematic screening can lead to identifying potentially at risk mothers. Since the risk is linked with experience of psychosocial stressors it seems that they might benefit from increased psychosocial support to prevent affective pathology.

## Background

During the postpartum period, women are vulnerable to clinical depression. There are two main types of postpartum depressive disorders: postpartum blues and postpartum depression (PPD).

Postpartum blues, also known as “the baby blues,” is a mild and brief mood problem. The prevalence rate for postpartum blues varies from 15.3% to 84% [1]. Symptoms usually begin three to 4 days after delivery and tend to resolve by day 12. The most frequent symptoms are mood swings with times of feeling anxious, irritable or tearful, poor appetite, and sleep problems [2, 3]. Symptoms are subtle and resolve spontaneously. However, up to 25% of patients with postpartum blues develop PPD [4].

The symptoms of PPD are identical to those of a major depressive episode but with a postpartum specifier [3]. The fifth edition of the Diagnostic and Statistical Manual (DSM-V) describes is as a major depression with peripartum onset, a diagnosis which can be applied if symptoms occur during pregnancy or in the 4 weeks following delivery [5]. However, many researchers extend the postpartum period beyond 4 weeks, some to six to 12 weeks [6], with the most frequent definition being 12 months after delivery [7, 8]. The main reason that PPD is considered a severe condition is that it leads to negative parenting practices, breastfeeding problems, and impaired child development [7, 9].

The data indicate that 10 to 20% of women experience a postpartum depressive episode [10]. In theory any mother could be affected by PPD, regardless of her age, number of other children or race [11]. Several studies have identified some risk factors for developing PPD. However, these studies have used only limited numbers of participants; therefore, the estimated prevalence of PPD varies greatly. Estimations of PPD prevalence depend mostly on the diagnostic method, the population examined, the time period studied, and the sampling bias [12]. There are no standardized diagnostic tools. The frequently used Edinburgh Postnatal Depression Scale [13] is a nonspecific tool for both depression and anxiety symptoms, and there is an inconsistency in the use of cut-off score. Moreover, fewer than half of PPD cases are diagnosed in clinical practice [14]. Therefore there is a need to improve case detection, identify risk populations, and implement evidence-based treatment [9].

The aim of this study is to examine depressive signs in a representative sample observed prospectively via the data collected in a longitudinal study and to identify sociodemographic and delivery risk factors. We try to verify the assumption that negative social events and delivery issues are related to the development of PPD as well as to confirm that personal history and family history of depression are risk factors. The main benefit of this study is that it uses a large amount of data obtained not only on depressive symptoms but also on the biological, socioeconomic, and environmental factors which can contribute to the development of PPD. Another benefit of this studied population is that mothers were enrolled in 1991 and 1992, shortly after the Velvet Revolution – the time of a change of regime from communism into democracy, which could be a time with a higher psychosocial burden. This could increase the risk of developing PPD. At that time, general knowledge about PPD was much lower than now which may have prevented overestimations in the self-administered questionnaires.

## Methods

### Sample

In this study, we used the data from the Czech part of the European Longitudinal Study of Pregnancy and Childhood (ELSPAC).

ELSPAC is a prospective longitudinal cohort study designed to investigate the effects of biological, psychosocial, economic, and environmental factors on pregnancy, delivery, and subsequent child development and health [15]. There are seven independent centers of ELSPAC: the United Kingdom, Isle of Man, Czech Republic, Slovakia, Ukraine, Greece, and Russia. The Czech ELSPAC study population has been defined as all pregnancies and births in two regions of the Czech Republic (Brno and Znojmo) between 1st April 1991 and 30th June 1992. Mothers were enrolled into the study in the period between an ultrasound examination at the 20th week of their pregnancy and the childbirth. Participating mothers were able to invite their partners to join the study as well. The enrolment of the Czech participants started in 1991 and the data collection period ended in 2011; the total sample size was 7589. In the following years, data were transferred into a comprehensive electronic database. The records consist of self-reported questionnaires from mothers, fathers, children, and their teachers, as well as from the mothers gynecologists and children’s pediatricians [16]. The primary objective of the Czech ELSPAC study was to evaluate the state of health of the birth cohorts. Our study used these data to assess sociodemographic and delivery risk factors for developing PPD. However, not all 7589 participants in the study completed questionnaires at all three time points we considered in our study (prenatal, 6 weeks postpartum, and 6 months postpartum). Therefore, we were unable to use their data for analysis because of the longitudinal design of our study. This natural drop-out was the only exclusionary criteria, we did not actively eliminate any other subjects. The number of subjects who completed questionnaires at all three time points was 3233.

### Edinburgh Postnatal Depression Scale

To identify PPD signs we used a Czech translation of the Edinburgh Postnatal Depression Scale (EPDS). The EPDS is a self-reporting questionnaire consisting of ten items validated for the postpartum population. The questionnaire takes approximately 5 minutes to complete [13].

We used the EPDS with a threshold of ten points, as was used in the original EPDS study [13]. However, the scale includes many questions about non-specific signs. For this reason, we conditioned the determination of depressive symptoms on a positive answer to question number eight, which refers to mood problems (a score of at least two means the participants have felt sad or miserable at least quite often). This condition was added to a threshold of ten points among the original ten questions.

### Variables

We tried to identify several risk variables. We included questions about the mother’s personal history and family history of depression (from the expectant mother’s maternal and paternal side). The next question was about whether the pregnancy was intentional. We included a question about whether the mother would describe herself as feeling unhappy about being pregnant. Further questions were if the mother was primiparous or multiparous, the gender of the child, the gestational maturity of the child, and the method of delivery. We included a question about newborns being transferred to intensive care units (ICU) to identify any possible links between serious newborn conditions and developing PPD. There was a question about the child’s nutrition– whether it was breastfed or formula fed. Finally, there were several questions about the mother – her age (we separated a group of adolescent mothers – under 18 years), highest level of education achieved, socioeconomic status (identified by whether the family is saving money) and whether the mother lives alone (without a partner).

The last variable we examined were psychosocial stressors. There were 35 questions regarding possible occurrences of psychosocial stressors, such as death or illness in close family members, signs of domestic violence, occupational or relationship problems, or financial difficulties. Questionnaires completed before childbirth examined the occurrence of these variables in the time period from the beginning of the pregnancy; questionnaires given 6 weeks after childbirth covered the second half of the pregnancy; and questionnaires completed at 6 months postpartum covered the period since childbirth. Participants marked the impact of each stressor on a scale from 0 to 4 points. The total points were a scale from 0 to 140 points making one variable: psychosocial stressors.

### Statistics

Standard descriptive statistics were used in the analyses. Categorical variables were described by absolute and relative frequencies. The means, supplemented by standard deviation or median with 5-95% percentile, were adopted for continuous variables.

Factors influencing maternal depression were analyzed using logistic regression. Results are presented as odds ratios (OR) supplemented by a 95% confidence interval. The Wald test was used to test the statistical significance of OR.

The results were considered statistically significant at the level of alpha <0.05 in all applied analyses. Analyses were performed using IBM SPSS 22.0.0 (IBM Corporation, 2013).

## Results

### Sample characteristics

**Fig. 1:**
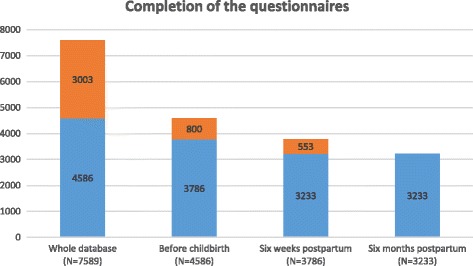
Completion of the questionnaires. Numbers of women who completed questionnaires at each time-point. Blue represents subjects who completed questionnaire at certain time-point as well as the following one. Orange represents women, who did not complete the following questionnaire (drop-outs)

This study used the Czech ELSPAC database, which includes 7589 mothers. A self-reported questionnaire during pregnancy and at least one questionnaire 6 weeks after childbirth were completed by 3768 mothers. More than 85% of them also completed a subsequent questionnaire at the sixth month after delivery (*N* = 3233). Also see Fig. 1 – Completion of the questionnaires (Table 1).

**Table 1:** Completion of the questionnaires

Whole database	*N* = 7589
Before childbirth	*N* = 4586
6 weeks postpartum	*N* = 3768
6 months postpartum	*N* = 3233

Questionnaires were completed 6 months after delivery by 3233 mothers with a mean age of 25.6 years (SD +/−4.8). Of these women, 36.1% had only a primary education (*N* = 1167), 43.9% had only finished high school (*N* = 1420), and 18.8% were university graduates (*N* = 609). The rest (*N* = 37) were marked as unknown. Roughly half of the mothers were multiparous (49.5%, *N* = 1588), and 48.9% were primiparous (*N* = 1570), the rest (*N* = 52) were marked as unknown. More than half of the children were male (51.9%, *N* = 1678), and 48.0% were females (*N* = 1553). Two children were marked as unknown gender (Table 2).

**Table 2:** Sample characteristics

Characteristics	Category	Results
Newborn’s gender	Male	1678 (51.9%)
	Female	1553 (48.0%)
	Unknown	2 (0.1%)
Birth order	Primiparous	1570 (48.9%)
	Multiparous	1588 (49.5%)
	Unknown	52 (1.6%)
Pregnancy term	Preterm (< 38 weeks)	83 (2.6%)
	Term	2397 (75.7%)
	Postmature (> 42 weeks)	10 (0.3%)
	Unknown	678 (21.4%)
Delivery route	Vaginal	2863 (88.6%)
	C-section	241 (7.5%)
	Other	59 (1.8%)
	Unknown	70 (2.2%)
Education of mothers	University	609 (18.8%)
	Secondary	1420 (43.9%)
	Primary	1167 (36.1%)
	Unknown	37 (1.1%)
Mean age of mothers		25.6 (4.8)

Data from all three time points are available from 3233 mothers. The data analysis was performed on this subsample of mothers. We used a threshold score of ten points in the EPDS and a positive answer to question number eight to identify the group of mothers with symptoms of a depressive episode. The prevalence of PPD in our sample was 12.8% during pregnancy (*N* = 414), 11.8% 6 weeks after childbirth (*N* = 380), and 10.1% 6 months after childbirth (*N* = 327) (Table 3).

There was a partial overlap between the three time points. Fewer than 2 % of mothers were depressed at all three time points; we detected no depressive signs at any time point in 75.5% of participants. Before childbirth, 414 (12.8%) of the mothers-to-be had signs of depression. Six weeks postpartum, 380 mothers (11.8%) had depressive signs; 130 of that 380 (34%) had also been depressed during the prenatal period and 175 of the 380 (46%) exhibited depressive signs only during this period. At 6 months postpartum, 327 mothers (10.1%) had signs of depression; 55 (16%) had also been depressed during the prenatal period and 6 weeks postpartum (Tables 3 and 4).

**Table 3:** Maternal depression

	Depression	Without depression
Before childbirth	414 (12.8%)	2819 (87.2%)
6 weeks postpartum	380 (11.8%)	2853 (88.2%)
6 months postpartum	327 (10.1%)	2906 (89.9%)

**Table 4:** Overlap in maternal depression

	N (%)
Whole time without depression	2441 (75.5%)
Prenatal depression only	215 (6.7%)
Postnatal depression only	175 (5.4%)
Depression only at 6 months after childbirth	128 (4.0%)
Prenatal and postnatal depression	75 (2.3%)
Depression postnatal and at 6 months after childbirth	75 (2.3%)
Prenatal depression and at 6 months after childbirth	69 (2.1%)
Depression at all three time points	55 (1.7%)

### Risk factors for prenatal maternal depression

Risk factors for prenatal depression were assessed based on questionnaire data collected in the period between an ultrasound examination at the 20th week of the pregnancy and the childbirth. We used univariate analysis to identify the following significant risk factors for developing prenatal depressive symptoms: personal history of depression (*N* = 167, OR = 2.7 (1.9; 3.9), *p* < 0.001), family history of depression of the expectant mother’s on both the maternal side (*N* = 553, OR = 1.7 (1.3; 2.2), *p* < 0.001) and paternal side (*N* = 154, OR = 1.9 (1.3; 2.9), *p* = 0.001), unintentional pregnancy (*N* = 1566, OR = 1.4 (1.2; 1.8), *p* = 0.001), feelings of unhappiness about being pregnant (*N* = 164, OR = 2.3 (1.6; 3.4), *p* < 0.001), only primary education (*N* = 1167, OR = 1.8 (1.3; 2.5), *p* < 0.001), mothers living alone (*N* = 88, OR = 3.0 (1.9; 4.8), *p* < 0.001) and psychosocial stressors (on a scale of 0–140 points, the increase of one point gained 9% (1.07; 1.10) greater chance of developing PPD; *p* < 0.001). The most frequent psychosocial stressors occurring at this period were thoughts that the mother might experience a spontaneous abortion, lowered income, and specialized testing for congenital anomalies. Good financial status, described as a family with monetary savings (*N* = 1274, OR = 0.7 (0.6; 1.0), *p* = 0.021), and male child gender (*N* = 1678, OR = 0.8 (0.7; 1.0), *p* = 0.047) were identified as protective factors against developing prenatal depressive signs. We found no contributing effects of the number of the children, secondary education, or mother’s age on prenatal depression.

Multivariate analysis identified the following significant risk factors for developing prenatal depressive symptoms: personal history of depression (*N* = 167, OR = 2.1 (1.2; 3.6), *p* = 0.008), family history of depression on the expectant mother’s paternal side (*N* = 154, OR = 1.8 (1.1; 3.1), *p* = 0.017) and psychosocial stressors (the increase of one point gained 7% (1.04; 1.09) more chance of developing PPD; *p* < 0.001). Male child gender (*N* = 1678, OR = 0.7 (0.5; 1.0), *p* = 0.022) and secondary education (*N* = 1420, OR = 0.6 (0.4; 0.9), *p* = 0.026) were identified as protective factors (Table 5).

**Table 5:** Risk factors for prenatal maternal depression

Risk factors		Frequency	Univariate analysis		Multivariate analysis	
			Odds ratio	*p*-value	Odds ratio	*p*-value
Personal or family history of depression	Personal history of depression	167 (5.2%)	2.7 (1.9; 3.9)	<0.001	2.1 (1.2; 3.6)	0.008
	Mother of the expectant mother had a personal history of depression	553 (19.0%)	1.7 (1.3; 2.2)	<0.001	1.2 (0.8; 1.7)	0.357
	Father of the expectant mother had a personal history of depression	154 (5.3%)	1.9 (1.3; 2.9)	0.001	1.8 (1.1; 3.1)	0.017
Factors regarding pregnancy	Unintentional pregnancy	1566 (48.7%)	1.4 (1.2; 1.8)	0.001	1.2 (0.9; 1.7)	0.173
	Mother felt unhappy about being pregnant	164 (5.2%)	2.3 (1.6; 3.4)	<0.001	1.5 (0.8; 2.7)	0.176
	Primiparous	1570 (48.6%)	1.0 (0.9; 1.3)	0.677	1.0 (0.7; 1.3)	0.798
	Gender of the child – male	1678 (51.9%)	0.8 (0.7; 1.0)	0.047	0.7 (0.5; 1.0)	0.022
	Mother under 18 years	41 (1.3%)	1.2 (0.5; 2.8)	0.725	0.9 (0.2; 4.3)	0.947
	Education – secondary^a^	1420 (44.4%)	1.3 (0.9; 1.8)	0.114	0.6 (0.4; 0.9)	0.026
	Education – primary^a^	1167 (36.5%)	1.8 (1.3; 2.5)	<0.001	0.8 (0.5; 1.1)	0.111
Factors regarding the time of questionnaire	Family savings^b^	1274 (56.9%)	0.7 (0.6; 1.0)	0.021	0.7 (0.5; 1.0)	0.056
	Mother living alone^b^	88 (2.7%)	3.0 (1.9; 4.8)	<0.001	1.6 (0.8; 3.5)	0.196
	Psychosocial stressors^c^	4.0 (0.0; 18.0)	1.09 (1.07; 1.10)	<0.001	1.07 (1.04; 1.09)	<0.001

^a^We used college education as a reference value

^b^Question was asked at 6 months postpartum

^c^This variable includes 35 questions regarding psychosocial stressors (the scale is 0-140 point), we stated median and a 5-95% percentile

### Risk factors for postnatal depression at 6 weeks postpartum

Risk factors for postnatal depression were assessed based on questionnaire data collected at 6 weeks after the delivery. In this period, we identified the following significant risk factors for developing depressive symptoms using univariate analysis: personal history of depression (*N* = 167, OR = 3.8 (2.7; 5.3), *p* < 0.001), family history of depression from both maternal (*N* = 553, OR = 1.8 (1.4; 2.4), *p* < 0.001) and paternal (*N* = 154, OR = 1.6 (1.1; 2.5), *p* = 0.026) sides, unintentional pregnancy (*N* = 1566, OR = 1.3 (1.1; 1.7), *p* = 0.009), feelings of unhappiness about being pregnant (*N* = 164, OR = 2.4 (1.6; 3.5), *p* < 0.001), mothers living alone (*N* = 88, OR = 3.7 (2.3; 5.9), *p* < 0.001), and psychosocial stressors (increase of one point in our stress scale made a 12% (1.10; 1.13) greater risk of developing PPD; *p* < 0.001). The most frequent psychosocial stressors occurring at this period were disagreements with partner, specialized testing for congenital anomalies, and lowered income. We found no significant association between PPD and the following factors: number of other children, child gender, gestational age, delivery route, newborn ICU transfers, breastfeeding, mother’s age, education, or financial status.

Multivariate analysis identified the following significant risk factors for developing postnatal depressive symptoms: personal history of depression (*N* = 167, OR = 2.7 (1.5; 4.9), *p* = 0.001), mothers living alone (*N* = 88, OR = 2.4 (1.1; 5.6), *p* = 0.033), and psychosocial stressors (an increase of one point made a 10% (1.07; 1.13) greater risk of developing PPD; *p* < 0.001) (Table 6).

**Table 6:** Risk factors for postnatal maternal depression at 6 weeks postpartum

Risk factor		Frequency	Univariate analysis		Multivariate analysis	
			Odds ratio	*p*-value	Odds ratio	*p*-value
Personal or family history of depression	Personal history of depression	167 (5.2%)	3.8 (2.7; 5.3)	<0.001	2.7 (1.5; 4.9)	0.001
	Mother of the expectant mother had a personal history of depression	553 (19.0%)	1.8 (1.4; 2.4)	<0.001	1.1 (0.7; 1.7)	0.659
	Father of the expectant mother had a personal history of depression	154 (5.3%)	1.6 (1.1; 2.5)	0.026	1.2 (0.7; 2.3)	0.508
Factors regarding pregnancy or delivery	Unintentional pregnancy	1566 (48.7%)	1.3 (1.1; 1.7)	0.009	1.2 (0.9; 1.8)	0.264
	Mother felt unhappy about being pregnant	164 (5.2%)	2.4 (1.6; 3.5)	<0.001	1.9 (1.0; 3.7)	0.051
	Primiparous	1570 (48.6%)	1.1 (0.9; 1.3)	0.626	1.1 (0.7; 1.5)	0.743
	Gender of the child – male	1678 (51.9%)	1.0 (0.8; 1.2)	0.724	1.2 (0.8; 1.7)	0.381
	Preterm birth (less than 38 weeks)	83 (3.3%)	0.8 (0.4; 1.7)	0.541	0.6 (0.1; 2.7)	0.534
	C-section	241 (7.6%)	1.4 (1.0; 2.0)	0.087	1.5 (0.8; 2.9)	0.241
	Newborn transfer to ICU	153 (4.9%)	0.8 (0.5; 1.4)	0.488	0.6 (0.2; 2.4)	0.519
	No breastfeeding	563 (18.4%)	1.2 (0.9; 1.6)	0.179	1.2 (0.8; 1.8)	0.392
	Mother under 18 years^a^	41 (1.3%)	0.8 (0.3; 2.3)	0.690	-	-
	Education – secondary^b^	1420 (44.4%)	0.9 (0.6; 1.2)	0.328	1.3 (0.8; 2.2)	0.242
	Education – primary^b^	1167 (36.5%)	0.9 (0.7; 1.3)	0.654	1.4 (0.9; 2.1)	0.146
Factors regarding the time of questionnaire	Family savings^c^	1274 (56.9%)	0.8 (0.6; 1.1)	0.192	0.8 (0.5; 1.1)	0.137
	Mother living alone^c^	88 (2.7%)	3.7 (2.3; 5.9)	<0.001	2.4 (1.1; 5.6)	0.033
	Psychosocial stressors^d^	4.0 (0.0; 17.0)	1.12 (1.10; 1.13)	<0.001	1.10 (1.07; 1.13)	<0.001

^a^Analysis not conducted due to small sample size

^b^We used college education as a reference value

^c^Question was asked at 6 months postpartum

^d^This variable includes 35 questions regarding psychosocial stressors (the scale is 0-140 point), we stated median and a 5-95% percentile

### Risk factors for maternal depression at 6 months postpartum

Risk factors for maternal depression at 6 months postpartum were acquired based on data collected 6 months after the childbirth. At this time point the following significant risk factors were identified in our study using univariate analysis: personal history of depression (*N* = 167, OR = 2.9 (2.0; 4.3), *p* < 0.001), family history of depression on the mother’s maternal side (*N* = 553, OR = 1.5 (1.2; 2.0), *p* = 0.003), feelings of unhappiness about being pregnant (*N* = 164, OR = 2.3 (1.5; 3.4), *p* < 0.001), mothers who opted not to breastfeed (*N* = 563, OR = 1.4 (1.0; 1.8), *p* = 0.025), mothers living alone (*N* = 88, OR = 4.0 (2.5; 6.4), *p* < 0.001), psychosocial stressors (an increase of one point resulted in a 13% (1.11; 1.15) greater chance of PPD; *p* < 0.001). The most frequent psychosocial stressors occurring at this period were lowered income, disagreements with partner, and if one of the mother’s children was ill. Primiparous mothers (*N* = 1570, OR = 0.8 (0.6; 1.0), *p* = 0.029) and mothers with family savings (*N* = 1274, OR = 0.7 (0.6; 1.0), *p* = 0.029) were identified as lower risk groups. We found no contributing effect of unintentional pregnancy, child gender, gestational age, delivery route, newborn transfers to ICU, mother’s age or education on depressive signs at 6 months postpartum.

Multivariate analysis identified the following significant risk factors: feelings of unhappiness about being pregnant (*N* = 164, OR = 2.5 (1.3; 4.7), *p* = 0.005) and psychosocial stressors (an increase of one point resulted in a 6% (1.04; 1.09) greater chance of PPD; *p* < 0.001). Primiparous mothers (*N* = 1570, OR = 0.6 (0.4; 0.9), *p* = 0.016) were at lower risk for developing PPD (Table 7).

**Table 7:** Risk factors for maternal depression at 6 months postpartum

Risk factor		Frequency	Univariate analysis			
			Odds ratio	*p*-value	Odds ratio	*p*-value
Personal or family history of depression	Personal history of depression	167 (5.2%)	2.9 (2.0; 4.3)	<0.001	1.5 (0.8; 2.9)	0.235
	Mother of the expectant mother had a personal history of depression	553 (19.0%)	1.5 (1.2; 2.0)	0.003	1.3 (0.9; 2.1)	0.196
	Father of the expectant mother had a personal history of depression	154 (5.3%)	1.5 (0.9; 2.4)	0.095	1.3 (0.7; 2.4)	0.431
Factors at the time of pregnancy or delivery	Unintentional pregnancy	1566 (48.7%)	1.2 (0.9; 1.5)	0.136	1.1 (0.7; 1.6)	0.806
	Mother felt unhappy about being pregnant	164 (5.2%)	2.3 (1.5; 3.4)	<0.001	2.5 (1.3; 4.7)	0.005
	Primiparous	1570 (48.6%)	0.8 (0.6; 1.0)	0.029	0.6 (0.4; 0.9)	0.016
	Gender of the child – male	1678 (51.9%)	1.0 (0.8; 1.2)	0.933	1.0 (0.7; 1.4)	0.863
	Preterm birth (less than 38 weeks)	83 (3.3%)	0.9 (0.4; 2.0)	0.854	0.3 (0.1; 1.7)	0.187
	C-section	241 (7.6%)	1.3 (0.9; 2.0)	0.175	1.1 (0.5; 2.3)	0.772
	Newborn transfer to ICU	153 (4.9%)	1.1 (0.7; 1.9)	0.626	1.7 (0.6; 5.0)	0.322
	No breastfeeding	563 (18.4%)	1.4 (1.0; 1.8)	0.025	1.5 (1.0; 2.3)	0.074
	Mother under 18 years	41 (1.3%)	0.2 (0.0; 1.6)	0.135	1.0 (0.1; 8.2)	0.997
	Education – secondary^a^	1420 (44.4%)	0.8 (0.6; 1.1)	0.167	0.8 (0.5; 1.4)	0.445
	Education – primary ^a^	1167 (36.5%)	1.1 (0.8; 1.5)	0.645	0.8 (0.5; 1.3)	0.359
Factors at the time of questionnaire	Family savings	1274 (56.9%)	0.7 (0.6; 1.0)	0.034	0.8 (0.5; 1.1)	0.154
	Mother living alone	88 (2.7%)	4.0 (2.5; 6.4)	<0.001	2.2 (0.9; 5.3)	0.075
	Psychosocial stressors^b^	5.0 (0.0; 20.0)	1.13 (1.11; 1.15)	<0.001	1.06 (1.04; 1.09)	<0.001

^a^We used college education as a reference value

^b^This variable includes 35 questions regarding psychosocial stressors (the scale is 0-140 point), we stated median and a 5-95% percentile

## Discussion

The objective of this study was to identify sociodemographic and delivery risk factors for developing PPD. We used the EPDS as a screening tool to identify women with depressive signs.

In our study we found the following risk factors: personal and family history of depression, socioeconomic factors, number of other children, child gender, breastfeeding and attitude towards pregnancy. No significant connection between PPD and delivery risk factors was found.

The most frequently described risk factor for developing PPD is a personal history of postpartum or nonpuerperal depressive episodes [17–24]. Llewellyn et al. [25] estimated a 50% to 62% increased risk of developing a depressive episode during pregnancy among women with a personal history of PPD. Women with a personal history of a major depressive episode are said to be at a 30% higher risk of developing PPD [26]. In our study, the association between personal history of a depressive episode and developing PPD was even higher – women were at 170% higher risk antepartum (OR = 2.7, *p* < 0.001), at 280% higher risk at 6 weeks postpartum (OR = 3.8, *p* < 0.001) and at 190% higher risk at 6 months postpartum (OR = 2.9, *p* < 0.001). In multivariate analysis, the OR were lower – 2.1 antepartum (*p* = 0.008), 2.7 at 6 weeks postpartum (*p* = 0.001) and nonsignificant at 6 months postpartum (OR = 1.5, *p* = 0.235). We also found an association between family history of depression and developing PPD which is consistent with numerous other studies [19–23, 27].

Many papers [11, 28–32] describe low socioeconomic status as a risk factor for developing PPD. Segre et al. [32] describes women with high income levels ($70,000 annually) to be at four times lower risk of developing PPD than women with low incomes ($10,000 annually). Limited financial means for raising an infant indicates a high amount of stress for the mother, which can lead to depression [20]. This is in concordance with our findings: women with family savings were less prone to develop prenatal depression (OR = 0.7, *p* = 0.021), or depressive signs in the 6 months postpartum (OR = 0.7, *p* = 0.034). However, this was not significant in the multivariate model.

We found a strong association in the univariate analysis between low levels of education and developing prenatal depression (OR = 1.8, *p* < 0.001). This result was also found in several other studies [33–36] that attribute this to a lower income being connected with a low level of education. In our study however the secondary education was found to be a protective factor (OR = 0.6, *p* = 0.026) in the prenatal period in a multivariate analysis. We found no association in the postpartum period.

Several studies describe the female gender of a child to be a risk factor for developing PPD [29, 31, 37, 38]. This was attributed to dissatisfaction with a child’s gender. However, other studies describe no association between PPD and a child gender [18, 19]. In our study, we found the male gender of a child to be a mild protective factor for developing antepartum depression (OR = 0.8, *p* = 0.047 in the univariate analysis, OR = 0.7, *p* = 0.022 in the multivariate analysis); no association was found in the postpartum period. This may suggest that dissatisfaction with a child’s gender was lowered after the delivery.

An unplanned or unwanted pregnancy may be a strong stressful event [39]. Kitamura et al. [38] describe a higher risk of antenatal depression among women with a negative attitude to a current pregnancy. Patel et al. [29] discovered planned pregnancy to be a very strong protective factor for developing PPD (OR = 0.3); this may be partially explained by the poverty of a researched territory. In our study we found unintentional pregnancy in the univariate model to be a mild risk factor antepartum (OR = 1.4, *p* = 0.001) and at 6 weeks postpartum (OR = 1.3, *p* = 0.009) with no association at 6 months postpartum. This may suggest that these women eventually accepted the role of motherhood. However, these associations were not confirmed in the multivariate model. If a mother felt unhappy about being pregnant, the risk of developing PPD was much higher in the univariate model (antepartum OR = 2.3, *p* < 0.001; at 6 weeks postpartum OR = 2.4, *p* < 0.001; at 6 months postpartum OR = 2.3, *p* < 0.001). The multivariate analysis confirmed this at 6 months postpartum (OR = 2.5, *p* = 0.005). This suggests an unwanted pregnancy is a stronger stressor than an unplanned pregnancy.

Other high risk group in our study were mothers without a partner (in the univariate analysis antepartum OR = 3.0, *p* < 0.001; at 6 weeks postpartum OR = 3.7, *p* < 0.001; at 6 months postpartum OR = 4.0, *p* < 0.001; in the multivariate model this was significant only at 6 weeks postpartum – OR = 2.4, *p* = 0.033), which was also described by Melo et al. [28]. Many other studies also found an association between PPD and the lack of family and social support [19, 21, 22, 40, 41].

Several studies [28, 42] described multiparity as a risk factor for PPD. Mathisen et al. [42] attribute this to a higher care burden and psychosocial stress. In our findings, there was no association between the number of other children and depression antepartum or postnatal, but we found primiparas to be at a lower risk at 6 months postpartum (in univariate analysis OR = 0.8, *p* = 0.029; in multivariate analysis OR = 0.6, *p* = 0.016).

In our study psychosocial stressors were also highly associated with developing PPD, which is in concordance with many other studies [11, 24, 43, 44] and emphasizes the relevance of stress in developing PPD.

Problems with breastfeeding are considered to be a strong risk factor for developing PPD. For example, McCoy et al. [17] found the relative risk of developing PPD when using artificial feeding techniques to be 2.04; Patel et al. [29] assessed the odds ratio of breastfeeding problems to be 3.1 in a sample of Indian women. Other studies showed roughly similar results [22, 41]. In our study, the univariate analysis suggested that women using artificial feeding techniques are at a higher risk for developing PPD at 6 months postpartum (OR = 1.4, *p* = 0.025), this was nonsignificant in the multivariate analysis (OR = 1.5, *p* = 0.074). The reason for this association varies across different papers. Misri et al. [45] report that most of their patients developed symptoms of PPD prior to the cessation of breastfeeding, suggesting that depressive symptoms lead to breastfeeding problems. The most frequently cited study, by Labbok [46], reported that in countries where exclusive breastfeeding is a norm the incidence of developing PPD peaks at 9 months; in countries prioritizing formula feeding the incidence peaks at 3 months after delivery. These results suggest that in the postpartum period, breastfeeding can be looked at as protective factor for developing PPD.

As in many other studies, we found no association between PPD and the gestational maturity of a child or the delivery route [17–19, 29, 47, 48]. No association was found between depressive signs in mothers and their newborns being transferred to ICU. This converges with findings of Hachem et al. [22],who describe newborn transfer to ICU as a risk factor of developing PPD (*p* = 0.025). However, this could be explained by the fact that they used an EPDS score only. Higher EPDS scores could also mean higher anxiety levels; it is not specific for PPD.

Troutman et al. [49] described adolescent mothers as a high risk group for developing PPD, with a prevalence of about 26%. This was not confirmed in our study or in many other studies describing lack of association with the age of mothers [17–20, 22, 24].

## Limitations

The biggest limitations are related to the ELSPAC study. It contains large amounts of data about the subjects, but there is a large drop-out rate among the study participants (mostly due to the high number of questions in each questionnaire), which introduces a possible selection bias. We were able to use the data from only 42.6% of the participants. The administration date of the first questionnaire was not very consistent – it was administered between the 20th week of pregnancy and the childbirth. Some of the questions included in the database also have methodological issues, such as the question about whether the family is saving money as an indicator of socioeconomic status.

Another limitation of our study is that it is a correlational study; it can only describe significant associations between sociodemographic and delivery risk factors, and it cannot determine the causality of these events, which would require further investigation.

We used the EPDS scale to detect depressive symptoms. It is considered to be a suboptimal tool for detecting clinically significant depressive syndrome and it cannot replace a systematic clinical interview. EPDS contains many items that are non-specific for depression, which can lead to high false positive rates. Moreover, there is no consensus on the depression detection based on EPDS – the EPDS cut-off score for identifying patients at risk for PPD varies through different studies. The original study recommends using a cut-off score of ten points or higher [13] which is still used in many countries [50–52]. According to some authors, an EPDS score of 12 points or higher is an accepted cut-off for recognizing patients at risk of PPD. [14, 22, 24, 53] Some authors prefer a threshold of 13 points [54, 55]. Other studies recommend lower scores – a threshold of nine points is commonly used [56–58], but some recommend a score of eight [59, 60] or seven points [61]. In our study we used the EPDS with a threshold of ten points, as was used in the original EPDS study [13]. With this methodology the incidence of depressive symptoms prenatal was 22.1%, it was 21,9% at 6 weeks postpartum and 18.4% at 6 months postpartum. This was higher than the expected incidence of PPD. We believe that a depressive mood is the most important sign of a depressive syndrome, therefore we conditioned positivity in our screening with a positive answer to item number eight referring to mood problems. In this way, we tried to increase the specificity of EPDS for depressive symptoms.

## Conclusion

The prevalence of PPD in a large epidemiological sample fluctuates between 10 and 12% before delivery, 6 weeks after delivery, and 6 months after delivery; however, only 2% of mothers were depressed at all three time points. The main risk factors for developing PPD identified as significant at all three time points were a personal history of previous depressive episodes and mothers who experience significant psychosocial stressors. We propose that mothers-to-be in these risk groups could benefit from screening for the presence of PPD. Since the risk is linked with experience of psychosocial stressors it seems that they might benefit from increased psychosocial support (social counseling, couple counseling, family and friend support etc.) to prevent affective pathology.

## Abbreviations

ELSPAC: European Longitudinal Study of Pregnancy and Childhood
EPDS: Edinburgh postnatal depression scale
ICU: Intensive care unit
OR: Odds ratio
PPD: Postpartum depression
